# Biliary Innate Immunity: Function and Modulation

**DOI:** 10.1155/2010/373878

**Published:** 2010-07-27

**Authors:** Kenichi Harada, Yasuni Nakanuma

**Affiliations:** Department of Human Pathology, Kanazawa University Graduate School of Medicine, Kanazawa 920-8640, Japan

## Abstract

Biliary innate immunity is involved in the pathogenesis of cholangiopathies in patients with primary biliary cirrhosis (PBC) and biliary atresia. Biliary epithelial cells possess an innate immune system consisting of the Toll-like receptor (TLR) family and recognize pathogen-associated molecular patterns (PAMPs). Tolerance to bacterial PAMPs such as lipopolysaccharides is also important to maintain homeostasis in the biliary tree, but tolerance to double-stranded RNA (dsRNA) is not found. In PBC, CD4-positive Th17 cells characterized by the secretion of IL-17 are implicated in the chronic inflammation of bile ducts and the presence of Th17 cells around bile ducts is causally associated with the biliary innate immune responses to PAMPs. Moreover, a negative regulator of intracellular TLR signaling, peroxisome proliferator-activated receptor-*γ* (PPAR*γ*), is involved in the pathogenesis of cholangitis. Immunosuppression using PPAR*γ* ligands may help to attenuate the bile duct damage in PBC patients. In biliary atresia characterized by a progressive, inflammatory, and sclerosing cholangiopathy, dsRNA viruses are speculated to be an etiological agent and to directly induce enhanced biliary apoptosis via the expression of tumor necrosis factor-related apoptosis-inducing ligand (TRAIL). Moreover, the epithelial-mesenchymal transition (EMT) of biliary epithelial cells is also evoked by the biliary innate immune response to dsRNA.

## 1. Introduction

Clarification of the molecular mechanisms of innate immunity and significance of innate immune responses to the pathogenesis of immune-mediated diseases as well as to the defense against infections has progressed steadily since the cloning of Tolls in drosophila and Toll-like receptors (TLRs) in mammals including humans [1, 2]. Innate immunity was initially thought to be limited to immunocompetent cells such as dendritic cells and macrophages, but epithelial cells also possess TLRs and proper innate immune systems. Liver and extrahepatic bile ducts consisting of hepatocytes and biliary epithelial cells (BECs) are also exposed to microorganisms and their components originating from the intestines via portal blood and duodenum. In the gastrointestinal tract, TLRs expressed in intestinal epithelial cells may be involved in innate immunity to maintain mucosal homeostasis and also the development of enterocolitis by producing inflammatory molecules [3]. Similar processes using TLRs may operate in the biliary tree. Human bile is sterile under normal conditions, but bacterial components such as lipopolysaccharide (LPS), lipoteichoic acid, and bacterial DNA fragments, known as pathogen-associated molecular patterns (PAMPs),   are detectable in normal and pathologic bile [4–7], and cultivable bacteria are also detectable in bile of patients with inflammatory biliary diseases [8–11], indicating that BECs are exposed to bacterial components under physiological as well as pathological conditions (Table 1). Although hepatocytes are usually infected by the hepatitis virus, no microorganisms showing BEC-specific tropism have been identified. The participation of microorganisms, however, in the etiology or pathogenesis of various cholangiopathies and biliary diseases has been reported or speculated. In this paper, we describe the biliary innate immune system, its association with the pathogenesis of cholangiopathy and biliary diseases, and finally a strategy for the attenuation of cholangiopathy, particularly cholangitis, by the regulation of innate immune responses.

## 2. Association with Biliary Innate Immunity in Biliary Diseases

Infectious agents have been implicated in the etiology or progression of cholangiopathies including cholangitis, bile duct loss, and lithiasis as a trigger or aggravating factor. Notably, several enterobacteria and viruses are speculated to be primary or secondary factor for primary biliary cirrhosis (PBC), primary sclerosing cholangitis, biliary atresia, hepatolithiasis, and chronic cholecystitis [4, 5, 12–16] (Table 1). 

### 2.1. Primary Biliary Cirrhosis (PBC)

PBC patients have an increased incidence of recurrent urinary tract infections compared to patients with other chronic liver diseases [17–19]. Recent findings also support an association between vaginal or urinary tract infections and PBC [20]. Furthermore, endotoxin and lipoteichoic acids abnormally accumulate in or around the intrahepatic bile ducts [7, 16, 21] and DNA of *Propionibacterium acnes* (*P. acnes*) was detected as a major clone in the granulomas of PBC patients (Table 1) [22]. Because these bacterial components, whether proteins or nucleic acids, act as PAMPs, the presence of PAMPs in bile or around bile ducts is known to induce a variety of inflammatory reactions and speculated to underlie the etiopathogenesis of the cholangiopathy in cases of PBC. 

 The major autoantigens against antimitochondrial antibodies (AMAs) in PBC are members of the 2-oxo-acid dehydrogenase complex (2-OADC), which includes the E2 subunit of the pyruvate dehydrogenase complex (PDC-E2) [23]. An immune response to intrahepatic BECs through 2-OADC-specific CD4^+^ helper T cells and CD8^+^ cytotoxic T cells is thought to be the major mechanism responsible for the immunological destruction of BECs in PBC and these T cells show molecular mimicry between human and bacterial PDC-E2 [24, 25]. Therefore, environmental factors such as microorganisms and xenobiotics are speculated to disrupt the self-tolerance to autoantigens as a specific intrahepatic BEC malfunction, supporting the forementioned role of PAMPs in the etiopathogenesis of PBC.

### 2.2. Biliary Atresia

Biliary atresia consists of a fetal type affecting 10–25% of patients and the more common perinatal type. The perinatal type is characterized by a progressive, inflammatory, and sclerosing cholangiopathy. The presence of several viruses including Reoviridae (type 3 reovirus and type C rotavirus) (Table 1) in liver tissue or affected bile duct specimens obtained from patients with biliary atresia during the Kasai procedure or a liver transplantation, has been demonstrated [13, 26–31], though conflicting results have been reported [13, 26, 28–30, 32]. Immunostaining for Mx proteins, which mediate an early innate immune response and are highly sensitive markers for type I interferon (IFN) activity, revealed that hepatocytes and intrahepatic bile ducts in biliary atresia are positive for Mx, suggesting the presence of viruses in hepatocytes and biliary epithelial cells of patients with biliary atresia [33]. Among these viruses, Reoviridae having a double-stranded RNA (dsRNA) genome, in particular, are characterized by epithelial tropism, and rotavirus type A is the most frequent etiological agent in cases of acute infantile diarrhea in young children. Moreover, the infection of newborn Balb/c-mice with Reoviridae including type A rhesus rotavirus (RRV) and type 3 reovirus (Abney) leads to a cholestasis and biliary obstruction resembling human biliary atresia [34, 35]. Therefore, it is likely that BECs are a target of these viruses which directly cause the cholangiopathy in cases of biliary atresia.

### 2.3. Hepatolithiasis

Hepatolithiasis is not a rare disease in East Asian countries including Japan and is characterized by the formation of stones and histologically “chronic proliferative cholangitis”. Bacterial infections in the biliary tree and cholestasis have been implicated as the major etiopathogenic factor for lithogenesis in patients with calcium-bilirubinate stones. *Escherichia coli* (*E. coli*) is the bacterium most frequently isolated, followed by several species shown to have *β*-glucuronidase (Table 1). Moreover, the presence of *Campylobacter* species-specific DNA has been demonstrated in bile samples and biliary mucosa specimens in cases of hepatolithiasis, by PCR (Table 1) [9]. These bacteria in the biliary epithelium are speculated to influence the occurrence and development of cholangitis and lithogenesis, though the mechanism of such an effect is still unclear.

## 3. Basic Mechanisms of Biliary Innate Immunity

**Figure 1:**
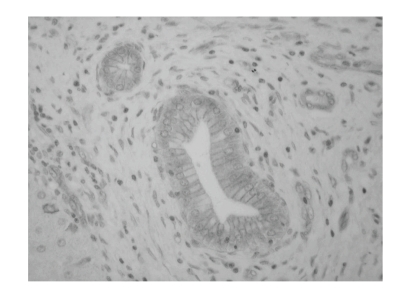
Representative expression pattern of TLR4 in interlobular bile ducts. Membranous (mainly lateral) in addition to weakly cytoplasmic expression is found in Primary biliary cirrhosis. Immunohistochemistry for TLR4.

BECs are immunologically  potent cells. The BECs of inflamed bile ducts  actively participate in the inflammation by secreting cytokines and expressing immune receptors. In addition to immunocompetent cells, epithelial cells including BECs recognize microbes and their constituents via a set of receptors, referred to as pattern-recognition receptors (PRRs). TLRs are the major epithelial PRRs recognizing PAMPs. Ten TLRs (TLR1 to TLR10) have been identified in humans, with TLR4 known to mediate inflammatory responses to LPS. In immunocompetent cells, the response to LPS is mediated by interaction with the TLR4 in conjunction with TLR4 accessory proteins MD-2 and CD14, triggering transduction of intracellular signals followed by the activation of TLR-associated adapter proteins, myeloid differentiation factor 88 (MyD88), and IL-1 receptor-associated kinase (IRAK)-1, leading to the activation of nuclear factor-*κ*B (NF-*κ*B) and then to the synthesis of antibiotics and proinflammatory cytokines. In contrast to bacterial PAMPs, dsRNA viruses such as Reoviridaes (reovirus and rotavirus) are recognized by TLR3, IFN-inducible helicase retinoic acid-induced protein I (RIG-I), and melanoma differentiation-associated gene-5 (MDA-5). The stimulation of these receptors by dsRNA transduces signals to activate the transcription factor interferon regulatory factor 3 (IRF3) as well as NF-*κ*B. Human and murine BECs possess at least TLR1-TLR5, related molecules (MD-2, MyD88, and IRAK-1), RIG-I, and MDA-5 [4, 36–38]. Moreover, immunohistochemistry has confirmed that TLR1-TLR5, MyD88, and IRAK-1 are distributed diffusely in the intrahepatic biliary tree of normal human liver, irrespective of anatomical levels [38] (Figure 1). In addition to the expression of these receptors, the responsiveness of BECs to the corresponding PAMPs is also found. For example, LPS binds to the surface of cultured BECs and induces the production of TNF-*α* mRNA in an NF-*κ*B-dependent manner [4]. Moreover, stimulation with polyinosinic-polycytidylic acid (poly(I:C), a synthetic analog of viral dsRNA) induces the activation of NF-*κ*B and IRF3 and the production of IFN-*β*1 and MxA as potent antiviral responses [37]. Therefore, BECs possess functional TLR signaling systems and participate in innate immunity.

## 4. Chemical Mediators Produced by a Biliary Innate Immune Response

Innate immunity provides defense against bacterial and viral infections. Therefore, as part of an innate immune response, several antibiotics are produced. Cytokines and chemokines are also produced in immunocompetent cells and play an important role in subsequent acquired immunity. Moreover, BECs have been shown to secrete polymeric immunoglobulin A, several antibiotics against bacteria (lactoferrin, lysozyme, and defensins) and viruses (IFN-*β*1 and MxA), cytokines, and chemokines on treatment with PAMPs, thereby contributing to biliary mucosal defense and subsequent acquired immunity [39–41].

### 4.1. Defensin

**Figure 2:**
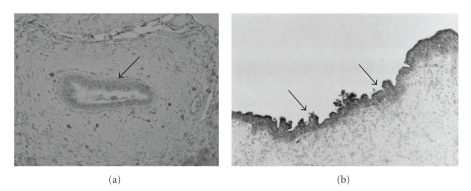
Immunohistochemical staining for human beta defensin (hBD)-1 (a) and hBD-2 (b). (a) Normal liver. Septal bile ducts are positive for hBD-1. (b) Extrahepatic biliary obstruction. Biliary epithelium of the intrahepatic large bile duct showing cholangitis strongly expresses hBD-2.

Defensins are antimicrobial peptides identified as key elements in innate immunity. Structurally, they are divided into *α*- and *β*-defensins. The *β*-defensin family is distributed in the epithelium of several organs, constituting an important barrier at mucosal surfaces. So far, human *β*-defensins (hBD-1 to -6) have been identified.  hBD-1 is constitutively expressed in cultured BECs and diffusely distributed in the cytoplasm of intrahepatic bile ducts irrespective of anatomical levels [42] (Figure 2). Moreover, because hBD-1 is constantly detectable in bile samples, hBD-1 it is believed to play a role in the constitutive antimicrobial defense of the hepatobiliary system [42]. This may be why biliary tract infections are rare and bile is sterile under physiological conditions, though the biliary tree is potentially exposed to enteric bacteria. In contrast, hBD-2 is not detected in BECs cultured without a stimulant, but *de novo* expression is found in LPS- or *E. coli*-treated BECs. *In vivo*, hBD-2 expression is restricted to the intrahepatic large bile ducts and peribiliary glands, in particular, showing cholangitis in extrahepatic biliary obstruction and hepatolithiasis (Figure 2) [42]. Because in these diseased livers, enteric bacteria are mostly cultivable in bile, the participation of bacteria-related cholangitis is closely associated with the hBD-2 expression in BECs. Therefore, hBD-1 plays a constitutive role in biliary antimicrobial defense, while hBD-2 expression is induced in response to local infections and may play a role in additional antimicrobial defenses.

### 4.2. Interleukin 8

IL-8 is a major cytokine of neutrophils, and functions not only as a chemoattractant of neutrophils, basophiles, and some populations of T cells, but also as an activator of neutrophils for releasing leukotrienes, activated oxygen, and neutrophil defensins. Bacteria or their products have been reported to induce the secretion of IL-8 from intestinal or gingival epithelial cells, and such cytokines and chemokines are speculated to be involved in epithelial cell damage during bacterial or fungal infections. Cultured human BECs express and release IL-8 in response to bacterial PAMPs including LPS [43]. IL-8 expression is found in proliferating bile ductules in various diseased livers and closely associated with neutrophilic infiltration [43]. Particularly cholangitis lenta defined as bile ductular proliferation, ductular cholestasis, and ductular epithelial damage, is also accompanied by a prominent neutrophilic infiltration [44]. Cholangitis lenta is usually encountered in septic conditions, so circulating infectious reagents such as bacterial toxins or products, cytokines, or chemokines are speculated to be involved in its pathogenesis. In a state of sepsis, particularly endotoxemia, BECs may secrete IL-8 and attract neutrophils to reactive bile ductules. IL-8 produced in bile ductular biliary epithelia is a potential target in the prevention of liver and biliary damage in diseased livers such as septic liver.

### 4.3. Fractalkine

Fractalkine (CX3CL1) is a chemokine with both chemoattractant and cell-adhesive functions and plays an important role in the migration of leukocytes to target sites under physiological as well as pathological conditions. Fractalkine is expressed in several epithelial cells under normal conditions and involved in the chemoattraction to the epithelial layer and adhesion of mononuclear cells expressing its receptor, CX3CR1. The fractalkine level elevated in serum concurrent with increased expression of CX3CR1 in liver-infiltrating mononuclear cells in patients with PBC [45], suggesting fractalkine to be critical for the generation and persistence of the portal lymphocytic infiltration in PBC. In fact, fractalkine is detectable in BECs of small bile ducts in normal and diseased livers, but it is increased in injured bile ducts of PBC [45]. Moreover, many CX3CR1-positive mononuclear cells infiltrate into portal tracts and most biliary intraepithelial lymphocytes in injured bile ducts are also positive for CX3CR1. Production of fractalkine in BECs is responsible for the chemoattraction of CX3CR1-positive lymphocytes into portal tracts and into biliary epithelia [45]. Recently, Shimoda et al. [46] demonstrated the significance of fractalkine and the precise mechanism of its production using populations of multiple intrahepatic cell types, including endothelial cells, liver sinusoidal endothelial cells, and BECs, to directly study the interaction of fractalkine-producing cells with liver-infiltrating mononuclear cells. Endothelial cells produced large amounts of fractalkine upon stimulation by PAMPs, though liver sinusoidal endothelial cells produced no fractalkine. Moreover, BECs also produced fractalkine; TLR3-stimulated BECs produced fractalkine after direct contact with TLR4-stimulated autologous monocytes. Innate immunity may lead to increased expression of fractalkine in the liver and contribute to the development of cholangiopathy in cases of PBC.

## 5. Tolerance

**Figure 3:**
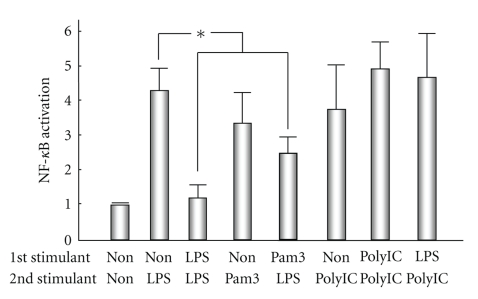
Induction of tolerance in cultured human biliary epithelial cells (BECs). BECs are pretreated with lipopolysaccharide (LPS, TLR4 ligand) or Pam_3_CSK_4 _(Pam3, TLR1/2 ligand) for 24 hours and subjected to another LPS challenge. Pretreatment with LPS and Pam_3_CSK_4_ significantly decreases NF-*κ*B activity in response to a subsequent LPS challenge. In contrast, the pretreatment with poly (I:C) (TLR3 ligand) or LPS does not inhibit NF-*κ*B's activation in response to a subsequent poly (I:C) challenge (* < .05).

The luminal surface of the biliary tree is continually exposed to PAMPs via bile and/or portal blood, but no inflammatory response is elicited in BECs. This lack of response to PAMPs, especially LPS, could be due to “endotoxin tolerance,” an important mechanism of maintaining the homeostasis of organs such as the intestines which have commensal bacterial flora and to avoid excessive tissue damage [47]. In addition to intestinal epithelial cells, BECs possess similar tolerance; treatment with LPS for 24 hours significantly induced tolerance to a subsequent exposure to LPS as assessed by measuring levels of NF-*κ*B activity and TNF-*α* mRNA production in cultured BEC cells (Figure 3) [48]. Moreover, pretreatment with Pam_3_CSK_4_ (TLR1/2 ligand) effectively induced tolerance to subsequent stimulation with LPS (TLR4 ligand) (Figure 3) [48]. This cross-tolerance has been demonstrated in monocytes and intestinal epithelial cells [47]. However, treatment with poly(I:C) (TLR3 ligand) significantly enhanced NF-*κ*B activity in fresh cultured BECs and pretreatment did not lead to tolerance to poly(I:C) (Figure 3) [49]. Levels of production of MxA and TRAIL were also preserved. Therefore, tolerance to a TLR3 ligand (poly(I:C)) is not found in BECs. 

**Figure 4:**
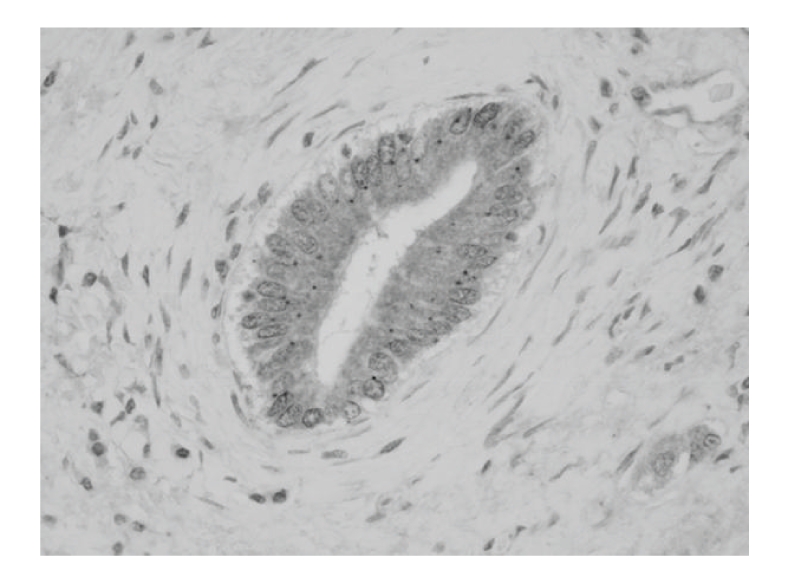
IRAK-M is constitutively expressed in the cytoplasm of septal bile ducts in normal liver. Immunohistochemistry for IRAK-M.

 In response to LPS, the structural complex formed by myeloid differentiation factor 88 (MyD88), IL-1 receptor-associated kinase (IRAK)-1, IRAK-4, and TNF receptor-associated factor 6 (TRAF6) induces a series of phosphorylation events, leading to the activation of nuclear transcription factors. IRAK-M plays a critical negative regulatory role in the signaling between MyD88 and IRAK-1 [50]. In LPS-tolerant cultured BECs, levels of the IRAK-M mRNA and protein were increased, implying that the expression of IRAK-M interferes with the association between IRAK-1 and MyD88 and is crucial to the LPS-induced tolerance of endotoxin in BECs [48]. Moreover, Pam_3_CSK_4_ as well as LPS induced IRAK-M expression in BECs [48], suggesting that the tolerance caused by the upregulation of IRAK-M expression is also associated with cross-tolerance to LPS induced by Pam_3_CSK_4_. Immunohistochemically, IRAK-M was constitutively expressed in the cytoplasm of BECs, irrespective of intrahepatic biliary levels (Figure 4). This finding suggests that the expression of IRAK-M is associated with hypo- or unresponsiveness to bacterial PAMPs in bile and/or portal flow. In contrast, although IRAK-M mRNA expression was upregulated by stimulation with dsRNA (TLR3 ligand), no tolerance to the dsRNA was found in cultured BECs. This is reasonable because the intracellular signaling of dsRNA-related receptors is a MyD88-independent pathway, that is, the dsRNA-related response is not affected by IRAK-M [51]. Moreover, the upregulation of IRAK-M expression on treatment with poly(I:C) is speculated to cause dsRNA-stimulated BECs to become tolerant to TLR2- and TLR3-related PAMPs including LPS. However, BECs are speculated to be in an entirely virus-free state and at the time of infection of a dsRNA virus, an innate response to dsRNA develops and continues in the presence of virus in the biliary tree, suggesting that the infection likely causes progressive destruction in the biliary epithelium without inducing tolerance.

## 6. Cholangiopathy Associated with Biliary Innate Immunity

### 6.1. Cell Death

BECs lining the biliary tree are tolerant of non-pathogenic commensal bacterial PAMPs so as to maintain the homeostasis of biliary innate immunity under physiological conditions. Even though biliary innate immunity is activated by pathological PAMPs and provides ‘danger signals' to the biliary tree, more effective negative mechanisms occur to avoid tissue damage. 

However, because innate immune tolerance of dsRNA is lacking in BECs, cell and tissue damage is found in the biliary innate immune response via TLR3 [49]. Stimulation with poly(I:C) induced the activation of NF-*κ*B and IRF-3, followed by the production of antiviral IFN-*β*1 [37] and also enhanced apoptosis via production of tumor necrosis factor-related apoptosis-inducing ligand (TRAIL) [37]. Moreover, in biliary atresia in which Reoviridae are speculated to be an etiological agent, BECs lining extrahepatic bile ducts diffusely and constantly expressed TLR3 and showed an enhancement of TRAIL and single-stranded DNA (ssDNA)-positive apoptosis as well as the activation of NF-*κ*B and IRF-3 and increased expression of an antiviral product, MxA [33, 37, 38]. Therefore, BECs not only directly participate in the antiviral innate immune response through the production of antiviral effectors to prevent viral replication by secreting antibiotics in response to dsRNA, but also play a role in the generation of apoptotic responses to infected cells. This additional mechanism concerning cell death in biliary innate immunity is directly associated with the pathogenesis of cholangiopathies in biliary atresia.

### 6.2. Epithelial-Mesenchymal Transition (EMT)

Fundamental to EMT is a loss of normal epithelial features such as cell-to-cell adhesion molecules and the gain of a mesenchymal phenotype [52]. Recently, the EMT of BECs has been speculated to be associated with periductal fibrosis and portal fibrosis in several chronic hepatobiliary diseases [53–56]. In biliary atresia, in particular, the mesenchymal markers vimentin, and S100A4 (also known as fibroblast-specific protein 1), and an essential transcription factor for EMT, Snail, are expressed but the biliary-type cytokeratin CK19 and the common epithelial marker E-cadherin are not, in BECs of extrahepatic bile ducts and peribiliary glands. The occurrence of EMT in the BECs of these anatomical biliary components is closely associated with the pathogenesis of sclerosing cholangiopathy in biliary atresia [56, 57]. As mentioned, although the biliary innate immune response to dsRNA reduces the viability of cultured human BECs via TRAIL-mediated apoptosis, the rate of cell death is approximately 30% [37]. The cells that evade apoptosis show a gradual loss of CK19 and E-cadherin, and increased expression of S100A4 and Snail, demonstrating the occurrence of biliary EMT. Because EMT confers resistance to apoptotic effects in fetal rat hepatocytes [58], biliary EMT is thought to be a survival mechanism and associated with an incomplete induction of apoptosis caused by the biliary innate immune response. 

 TGF-*β*1 and basic fibroblast growth factor (bFGF) are the major inducers of EMT and TGF-*β*1 plays an important role in the initiation and progression of liver fibrosis [59, 60]. Moreover, because expression of the TGF-*β*1 pseudoreceptor, bone morphogenic protein and activin membrane-bound inhibitor (Bambi), is decreased by an innate immune response and consequently, susceptibility to TGF-*β*1 is increased, loss of Bambi and upregulation of the TGF-*β* receptor are also speculated to be inducers of EMT [61]. Cultured human BECs constantly express TGF-*β*1, its receptor TGF*β*R1, and the bFGF receptor (FGFR1) [54, 57]. However, because Bambi is also expressed in BECs, the induction of EMT is likely inhibited by the effect of Bambi. Treatment with poly (I:C) gradually decreases and increases the expression of Bambi and bFGF, respectively, and stimulation with bFGF quickly induces a reduction in the level of Bambi. Therefore, the biliary innate immune response to dsRNA could increase susceptibility to TGF-*β*1, and both TGF-*β* and bFGF play important roles in the biliary EMT induced by a dsRNA-related innate immune response.

### 6.3. Chronic Inflammation

Recently, in addition to Th1 and Th2 cells, a third type of pathogenic helper T cell, the Th17 cell, and its association with the chronic inflammation of autoimmune diseases, has been noted [62]. Human Th17 cells are characterized by the production of interleukin (IL)-17 (IL-17A and IL-17F) and differentiate from naïve T cells (Th0). Th17 cells are part of the mucosal host defense system and have a major role in protection against bacterial infections. Moreover, with the discovery that Th17 cells are also involved in the pathogenesis of chronic inflammatory disorders including models of some autoimmune diseases, there has been intense interest in the relative contributions of Th17 and Th1/Th2 cells to the pathogenesis of these diseases. In liver, IL-17-positive cells identified as Th17 cells are mainly present at the interface of inflammed portal tracts in cases of PBC and CVH-C, and also, in PBC, accumulated around damaged interlobular bile ducts [63]. Th17 cells are associated with interface hepatitis in chronic liver diseases. Moreover, the Th17-related peribiliary cytokine milieu is enhanced in PBC and implicated in the histogenesis of the sustained cholangitis of PBC.

In human Th17 cells, IL-6 and IL-1*β* are required for differentiation [64], while IL-23 is necessary for maintaining or stabilizing cellular functions and survival, but not differentiation [62]. Bacterial PAMPs including LPS cause the production of Th17-inducible cytokines (IL-6 and IL-1*β*) and a Th17-maintaining cytokine (IL-23) in cultured BECs [63]. Moreover, BECs lining damaged bile ducts in PBC, express IL-6, IL-1*β*, and IL-23 p19 [63]. The biliary innate immune response to bacterial components involves the production of Th17-inducible and -maintaining cytokines in BECs and also the differentiation into Th17 cells of periductal dendritic cells and macrophages. Biliary innate immunity plays a role in the induction and maintenance of Th17 cells in the periductal area in cases of PBC.

## 7. Modulation of Biliary Innate Immunity as a Therapeutic Strategy

**Figure 5:**
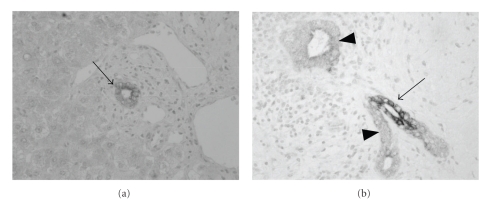
Immunohistochemistry for peroxisome proliferator-activated receptor *γ* (PPAR*γ*). (a) Normal liver. PPAR*γ* is expressed in the cytoplasm of bile ducts (arrow). (b) Primary biliary cirrhosis (PBC). Damaged bile ducts (arrowhead) show reduced expression of PPAR*γ*, though evidently positive biliary cells (arrow) also remain.

Therapeutic strategies have been proposed for the modulation of hepatic innate immunity, but rarely for biliary innate immunity. Peroxisome proliferator-activated receptor *γ* (PPAR*γ*) is a nuclear receptor involved in regulating adipocyte differentiation and also antiinflammatory activities [65]. The activation of PPAR*γ* by its ligands is shown to inhibit the expression of proinflammatory cytokines such as TNF-*α*, the induction of which is mediated via NF-*κ*B [66]. In liver, PPAR*γ* is constitutively expressed in intrahepatic bile ducts, irrespective of the anatomical level (Figure 5), and may relate to the maintenance of biliary homeostasis and absence of inflammatory reactions by attenuating inflammatory signals in BECs to PAMPs [67]. In PBC liver, PPAR*γ* expression is significantly downregulated in the affected bile ducts (Figure 5), indicating an increased susceptibility to PAMPs. IL-4 (Th2 cytokine) and IFN-*γ* (Th1 cytokine) up and downregulate PPAR*γ* expression, respectively, in cultured human BECs. An unique cytokine milieu is associated with the reduction in PPAR*γ* expression in the affected bile ducts of PBC liver [67].

**Figure 6:**
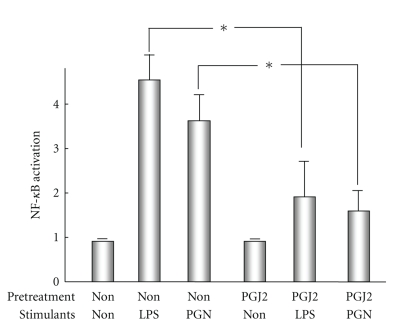
Effect of the peroxisome proliferator-activated receptor *γ* (PPAR*γ*) ligand, 15d-PGJ2, on lipopolysaccharide (LPS, TLR4 ligand)- and peptidoglycan (PGN, TLR2 ligand)-induced NF-*κ*B activation in cultured human biliary epithelial cells (BECs). BECs are pretreated in the presence or absence of 15d-PGJ2 (20 *μ*M) before stimulation with LPS or peptidoglycan. Pretreatment with 15d-PGJ2 significantly prevents PAMP-induced NF-*κ*B activation (* < .05).

Several PPAR*γ* ligands have been identified, including the prostaglandin D metabolite 15-deoxy-Δ^12,14^-prostaglandin J2 (15d-PGJ2) and thiazolidinedione derivatives. 15d-PGJ2 functions as an endogenous ligand for PPAR*γ* and attenuates the activation of NF-*κ*B by preventing the phosphorylation of its inhibitor protein (I-*κ*B). In cultured human BECs, 15d-PGJ2 treatment attenuates PAMP (LPS or peptidoglycan)-induced NF-*κ*B activation and also TNF-*α* production (Figure 6). PPAR*γ* ligands provide protection against biliary inflammation in PBC, but 15d-PGJ2 inhibited NF-*κ*B's activation independent of PPAR*γ* [67, 68]. In fact, a PPAR*γ* antagonist (GW9662) partially blocked the inhibitory effects of 15d-PGJ2 on NF-*κ*B activity. Therefore, 15d-PGJ2 is expected to be effective in the antiinflammatory treatment of bile ducts with reduced as well as preserved PPAR*γ* expression in PBC. Because PPAR*γ* is a key immunomodulatory molecule, a reduction in its expression in the bile ducts of PBC liver may be important to the immunopathogenesis of chronic cholangitis. Therefore, immunosuppression using PPAR*γ* ligands may help to reduce bile duct damage in PBC.

## 8. Conclusion

Biliary innate immunity consisting of an organ-specific system is important for the mucosal immunity in intrahepatic and extrahepatic bile ducts. Biliary innate immunity is surely associated with the pathogenesis of biliary diseases as well as the defense against microbial infections. The molecular mechanisms involved have recently been clarified. Targeting of NF-*κ*B is thought to be a potential therapeutic strategy in various cells, but TLR signaling-specific molecules are also likely to be suitable molecular targets. Further translational research concerning the regulation of biliary innate immunity is needed.

## Figures and Tables

**Table 1:** Bacteria and viruses possible etiological of biliary diseases.

Primary biliary cirrhosis
(i) Detection of microorganisms
(a) lipopolysaccharide (LPS)
(b) lipoteichoic acid
(c) *Helicobacter *
(d) *β*-retrovirus
(e) *P. acnes *
(ii) Molecular mimicry between human and microbial PDC-E2
(a) *E. coli *
(b) *Mycobacterium *
(c) *Novosphingobium *
(d) *Lactobacillus *
(e) Chlamydia
(iii) Biliary atresia
(a) Reovirus
(b) Rotavirus
(c) cytomegalovirus (CMV)
(d) adenovirus
(e) enterovirus
(f) Ebstein-Barr virus (EBV)
(iv) Primary sclerosing cholangitis
(a) *Helicobacter *
(b) *α* *-hemolytic streptococcus *
(v) Hepatolithiasis
(a) *Escherichia coli* (*E. coli*)
(b) *Klebsiella *
(c) *Streptococcus *
(d) *Pseudomonas *
(e) *Bacteroides *
(f) *Clostridium *
(g) *Campylobacter *
